# Poly[diaqua­[μ-1,4-bis­(1*H*-imidazol-1-yl)benzene-κ^2^ *N* ^3^:*N* ^3′^](μ-fumarato-κ^2^ *O* ^1^:*O* ^4^)nickel(II)]

**DOI:** 10.1107/S1600536812038895

**Published:** 2012-09-22

**Authors:** Chang-Xin Bian, Xiao-Qiang Yao, Yu-Min Song

**Affiliations:** a College of Chemistry and Chemical Engineering, Northwest Normal University, Lanzhou 730070, People’s Republic of China

## Abstract

In the title compound, [Ni(C_4_H_2_O_4_)(C_12_H_10_N_4_)(H_2_O)_2_]_*n*_, the Ni^II^ ion has a distorted octa­hedral coordination geometry. The asymmetric unit is composed of an Ni^2+^ ion, located on a twofold rotation axis, one half of a 1,4-bis­(1*H*-imidazol-1-yl)benzene (BIMB) ligand and one half of a fumarte (fum^2−^) dianion, both ligands being located about inversion centers, and a coordinating water mol­ecule. The Ni^II^ ions are linked by two BIMB ligands and two fum^2−^ dianions, forming a four-connected layered structure parallel to (010) with a 4^4^-sql topology. Within each layer, there are rhombic grids with dimensions of *ca* 13.5 × 9.0 Å and approximate angles of 109 and 70°. The crystal packing features a two-dimensional → two-dimensional parallel/parallel interpenetration in which one undulating layer is catenated to another equivalent one, forming a new bilayer. Moreover, the entangled two-dimensional layers are connected by O—H⋯O and C—H⋯O hydrogen bonds, generating a three-dimensional structure.

## Related literature

For multi-dimensional coordination polymers and their applications, see: Batten & Robson (1998 ▶); Carlucci *et al.* (2003*a* ▶,*b* ▶); Moulton & Zaworotko (2001 ▶); Sun *et al.* (2006 ▶); Wu *et al.* (2011 ▶); Bu *et al.* (2004 ▶). For their potential applications in electron transfer and drug delivery, see: Harriman & Sauvage (1996 ▶); Raymo & Sauvage (1999 ▶). For the structures of some related compounds, see: Chen *et al.* (2010 ▶); Li *et al.* (2012 ▶); Bu *et al.* (2004 ▶).
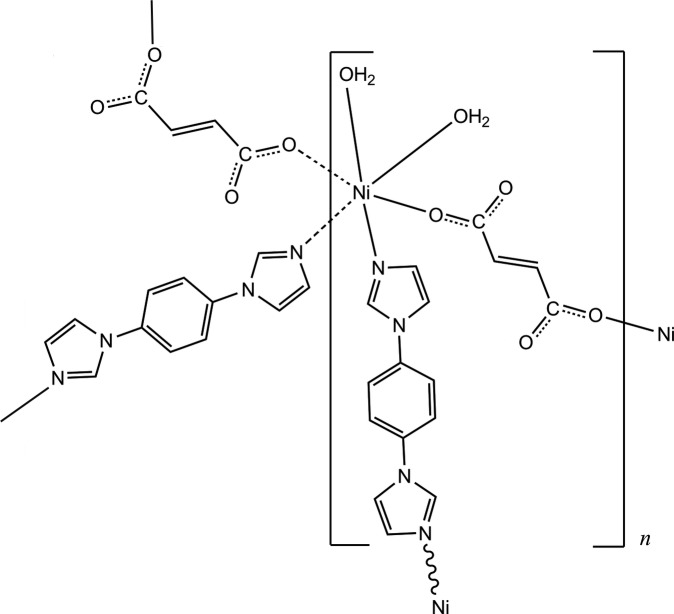


## Experimental

### 

#### Crystal data

[Ni(C_4_H_2_O_4_)(C_12_H_10_N_4_)(H_2_O)_2_]
*M* *_r_* = 419.04
Orthorhombic, 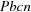

*a* = 11.2806 (4) Å
*b* = 16.3703 (7) Å
*c* = 9.0253 (3) Å
*V* = 1666.67 (11) Å^3^
*Z* = 4
Mo *K*α radiation
μ = 1.21 mm^−1^
*T* = 296 K
0.23 × 0.22 × 0.20 mm

#### Data collection

Bruke APEXII CCD area-dector diffractometer
Absorption correction: multi-scan (*SADABS*; Sheldrick, 1996 ▶) *T*_min_ = 0.768, *T*_max_ = 0.794
8512 measured reflections
2108 independent reflections
1827 reflections with *I* > 2σ(*I*)
*R*_int_ = 0.019

#### Refinement

*R*[*F* ^2^ > 2σ(*F* ^2^)] = 0.030
*wR*(*F* ^2^) = 0.093
*S* = 1.08
2108 reflections
123 parameters
2 restraints
H-atom parameters constrained
Δρ_max_ = 0.36 e Å^−3^
Δρ_min_ = −0.48 e Å^−3^

### 

Data collection: *APEX2* (Bruker, 2004 ▶); cell refinement: *SAINT* (Bruker, 2003 ▶); data reduction: *SAINT*; program(s) used to solve structure: *SHELXS97* (Sheldrick, 2008 ▶); program(s) used to refine structure: *SHELXL97* (Sheldrick, 2008 ▶); molecular graphics: *SHELXTL* (Sheldrick, 2008 ▶) and *DIAMOND* (Brandenburg, 2010 ▶); software used to prepare material for publication: *SHELXTL* and *publCIF* (Westrip, 2010 ▶).

## Supplementary Material

Crystal structure: contains datablock(s) I, global. DOI: 10.1107/S1600536812038895/su2481sup1.cif

Structure factors: contains datablock(s) I. DOI: 10.1107/S1600536812038895/su2481Isup2.hkl

Additional supplementary materials:  crystallographic information; 3D view; checkCIF report

## Figures and Tables

**Table 1:** Hydrogen-bond geometry (Å, °)

*D*—H⋯*A*	*D*—H	H⋯*A*	*D*⋯*A*	*D*—H⋯*A*
O3—H3*Y*⋯O2^i^	0.85	1.96	2.7033 (18)	146
O3—H3*X*⋯O2^ii^	0.85	2.03	2.8361 (18)	159
C3—H3⋯O2^ii^	0.93	2.49	3.360 (2)	155

Symmetry codes: (i) 
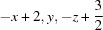
; (ii) 
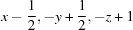
.

**Fig. 1.**
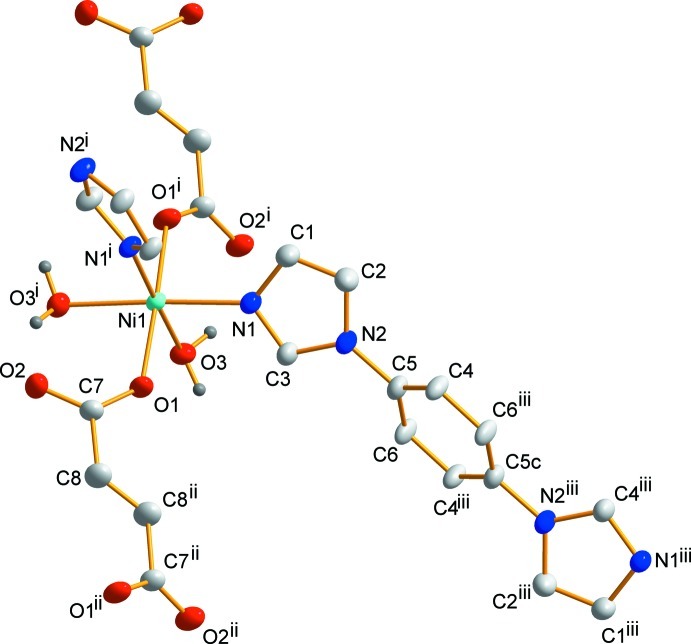
The molecular structure of the title compound, with atom numbering. The displacement ellipsoids are drawn at the 50% probability level [H atoms have been omitted for clarity; symmetry codes: (i) 1 – x, y, 0.5 – z; (ii) 1 – x, y, -0.5 – z; (iii) 2 – x, –y, –z].

**Fig. 2.**
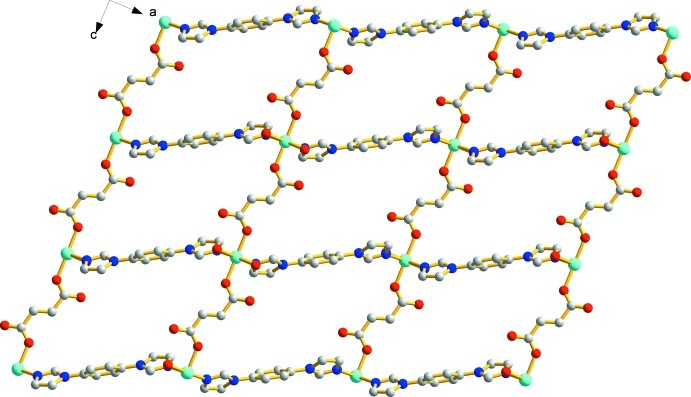
A view of the two-dimensional undulated 44-sql layer of the title compound.

**Fig. 3.**
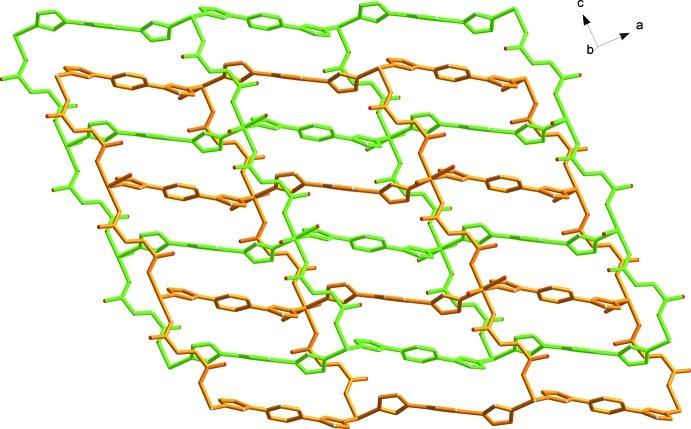
A view of the two-fold parallel polycatenation of the two-dimensional layers in the crystal structure of the title compound.

**Fig. 4.**
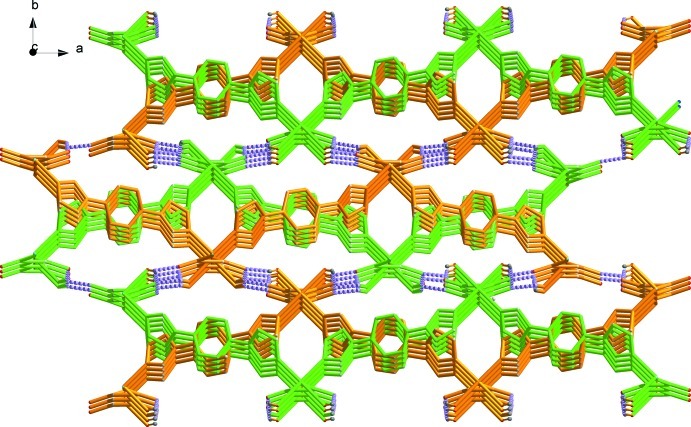
A view of the entangled two-dimensional layers that extended to a three-dimensional structure via O–H···O hydrogen bonds in the crystal structure of the title compound.

## supplementary crystallographic information

### Comment

Entanglement, one of the ubiquitous phenomena in nature, has received considerable attention due to their intrinsic aesthetic architectures (Bu *et al.*, 2004; Carlucci *et al.*, 2003*a*; Wu *et al.*, 2011) and potential applications (Sun *et al.*, 2006; Moulton & Zaworotko, 2001). Many structurally interesting entangled structures, such as polyrotaxane, polycatenation, polythreading, have been discussed in detail by (Batten *et al.*, 1998; Carlucci *et al.*, 2003*b*). Polycatenation as a type of interesting networks of entangled systems has attracted much attention for their potential application in energy of electron transfer and drug delivery (Harriman & Sauvage, 1996; Raymo & Sauvage, 1999). Herein, we report on the crystal structure of a Ni^II^ coordination polymer built from linear BIMB and fum^2-^ ligands, which features a two-dimensional → two-dimensional parallel/parallel polycatenation network.

The asymmetric unit of the title compound contains half a Ni^II^ ion located on a two-fold rotation axis, half a fum^2-^ dianion and half a BIMB ligand both located about inversion centers, and a coordinated water molecule. Each Ni^II^ ion is coordinated by two water molecules, two different carboxylate O atoms from two different fum^2-^ dianions and by two N atoms from two different BIMB ligands, and has a distorted octahedral geometry (Fig. 1).

It is interesting to note that the maleic acid (hydrolysis product of maleic anhydride) is converted into fumaric acid on the self-assembly of the title compound. This is probably because *trans*-fumaric has a higher thermal stability than *cis*-maleic acid.

In the crystal, each Ni^II^ ion is connected by two BIMB ligands and two fum^2-^ ligands to form an infinite two-dimensional puckered sheet with rhombic grids (Fig. 2). Within each layer, the rhombic grids have dimensions of ca. 13.5 Å × 9.0 Å with angles of of ca. 109.60 and 70.40° (defined by Ni···Ni distances and Ni···Ni···Ni angles). The large size of the grids in two adjacent layers allow a two-dimensional → two-dimensional parallel/parallel polycatenation to occur (Fig. 3). From a topological perspective, each Ni^II^ ion can be regarded as a four-connected node, thus this two-dimensional network can be assigned to the 4^4^-sql topology.

Moreover, the entangled two-dimensional layers are further connected by O–H···O hydrogen bonds to generate a three-dimensional structure (Fig. 4).

The structure of a similar Ni^II^ coordination polymer assembled by BIMB ligand and adipic acid has been described by (Chen *et al.*, 2010). However, compared with the title compound, the adipic acid is a longer spacer length and more flexible, and crystallizes in the lower symmetry triclinic space group *P*1 rather than orthorhombic space group *Pbcn* for the title compound with the short fumarate spacer.

Another relevant example reported by (Bu *et al.*, 2004) is a Zn^II^ coordination polymer (Li *et al.* 2012). Like the title complex, it is also built from BIMB and fum^2-^ ligands. However, the difference in the metal center results in an interesting 5-fold interpenetrated three-dimensional framework based on a diamondoid topology.

In summary, we have synthesized a Ni^II^ coordination polymer by the hydrothermal reaction of Ni(NO_3_)_2_ with H_2_fum and BIMB ligands, which features a two-dimensional → two-dimensional parallel/parallel polycatenation network. On comparing with two relevant complexes based on the BIMB ligand, we found that the coordination geometry of the central metal ions and the flexibility of the auxiliary carboxylate ligands indeed have a significant effect on the architecture of the target complexes.

### Experimental

A mixture of 1,4-Bis(1-imidazolyl)benzene (BIMB) (0.032 g, 0.15 mmol), maleic anydride (0.015 g, 0.15 mmol) and Ni(NO_3_)_2_ (0.045 g, 0.25 mmol) in *N*,*N*'-dimethylformamide (DMF) (4 ml) and H_2_O (2 ml) was placed in a Teflon-lined stainless steel vessel and heated at 363 K for 3 days. On cooling to room temperature green block-like single crystals suitable for X-ray diffraction were obtained [70% yield (based on BIMB ligand)]. Anal. Calcd for C_16_H_16_N_4_O_6_Ni: C, 45.86; H, 3.85; N, 13.37%. Found: C, 45.93; H, 3.87; N, 13.41%. Spectroscopic data for the title compound are given in the archived CIF.

### Refinement

The water H atoms were located in a difference Fourier map and included as riding atoms, with O—H = 0.85 and *U*_iso_(H) = 1.5Ueq(O). The C-bound H atoms were placed in calculated positions and treated as riding: C—H = 0.93 Å with *U*_iso_(H) = 1.2U_eq_(C).

### Crystal data

**Table:**

[Ni(C_4_H_2_O_4_)(C_12_H_10_N_4_)(H_2_O)_2_]	*F*(000) = 864
*M**_r_* = 419.04	*D*_x_ = 1.670 Mg m^−^^3^
Orthorhombic, *P**b**c**n*	Mo *K*α radiation, λ = 0.71073 Å
Hall symbol: -P 2n 2ab	Cell parameters from 4217 reflections
*a* = 11.2806 (4) Å	θ = 2.5–28.4°
*b* = 16.3703 (7) Å	µ = 1.21 mm^−^^1^
*c* = 9.0253 (3) Å	*T* = 296 K
*V* = 1666.67 (11) Å^3^	Block, green
*Z* = 4	0.23 × 0.22 × 0.20 mm

### Data collection

**Table:**

Bruke APEXII CCD area-dector diffractometer	2108 independent reflections
Radiation source: fine-focus sealed tube	1827 reflections with *I* > 2σ(*I*)
Graphite monochromator	*R*_int_ = 0.019
CCD rotation images, thin slices scans	θ_max_ = 28.5°, θ_min_ = 2.2°
Absorption correction: multi-scan (*SADABS*; Sheldrick, 1996)	*h* = −13→15
*T*_min_ = 0.768, *T*_max_ = 0.794	*k* = −18→21
8512 measured reflections	*l* = −12→12

### Refinement

**Table:**

Refinement on *F*^2^	Primary atom site location: structure-invariant direct methods
Least-squares matrix: full	Secondary atom site location: difference Fourier map
*R*[*F*^2^ > 2σ(*F*^2^)] = 0.030	Hydrogen site location: inferred from neighbouring sites
*wR*(*F*^2^) = 0.093	H-atom parameters constrained
*S* = 1.08	*w* = 1/[σ^2^(*F*_o_^2^) + (0.047*P*)^2^ + 1.0911*P*] where *P* = (*F*_o_^2^ + 2*F*_c_^2^)/3
2108 reflections	(Δ/σ)_max_ < 0.001
123 parameters	Δρ_max_ = 0.36 e Å^−^^3^
2 restraints	Δρ_min_ = −0.48 e Å^−^^3^

### Special details

**Table:**

Experimental. Spectroscopic data for the title compound :IR (KBr, cm^-1^): 3380m, 3133m, 1564s, 1533s, 1385s, 1307w, 1269w, 1130w, 1195w, 1074m, 970w, 880w, 829m, 751m, 682w, 656w, 534w, 495w.
Geometry. All e.s.d.'s (except the e.s.d. in the dihedral angle between two l.s. planes) are estimated using the full covariance matrix. The cell e.s.d.'s are taken into account individually in the estimation of e.s.d.'s in distances, angles and torsion angles; correlations between e.s.d.'s in cell parameters are only used when they are defined by crystal symmetry. An approximate (isotropic) treatment of cell e.s.d.'s is used for estimating e.s.d.'s involving l.s. planes.
Refinement. Refinement of *F*^2^ against ALL reflections. The weighted *R*-factor *wR* and goodness of fit *S* are based on *F*^2^, conventional *R*-factors *R* are based on *F*, with *F* set to zero for negative *F*^2^. The threshold expression of *F*^2^ > σ(*F*^2^) is used only for calculating *R*-factors(gt) *etc*. and is not relevant to the choice of reflections for refinement. *R*-factors based on *F*^2^ are statistically about twice as large as those based on *F*, and *R*- factors based on ALL data will be even larger.

### Fractional atomic coordinates and isotropic or equivalent isotropic displacement parameters (Å^2^)

**Table:**

	*x*	*y*	*z*	*U*_iso_*/*U*_eq_	
C1	0.85967 (16)	0.48653 (12)	0.8087 (2)	0.0337 (4)	
H1	0.9127	0.5069	0.8785	0.040*	
C2	0.76108 (18)	0.52549 (13)	0.7609 (2)	0.0353 (4)	
H2	0.7344	0.5768	0.7899	0.042*	
C3	0.77741 (16)	0.40688 (11)	0.6505 (2)	0.0294 (4)	
H3	0.7616	0.3627	0.5889	0.035*	
C4	0.57558 (18)	0.56487 (12)	0.5293 (3)	0.0409 (5)	
H4	0.6266	0.6082	0.5487	0.049*	
C5	0.60153 (15)	0.48748 (11)	0.5802 (2)	0.0290 (4)	
C6	0.52718 (19)	0.42289 (12)	0.5509 (3)	0.0408 (5)	
H6	0.5461	0.3709	0.5851	0.049*	
C7	1.08521 (15)	0.29993 (11)	0.43626 (19)	0.0266 (3)	
C8	1.05530 (18)	0.30159 (14)	0.2748 (2)	0.0348 (4)	
H8	1.1169	0.3027	0.2064	0.042*	
N1	0.86960 (13)	0.41195 (10)	0.73829 (16)	0.0261 (3)	
N2	0.70833 (13)	0.47384 (9)	0.66080 (18)	0.0288 (3)	
Ni1	1.0000	0.323481 (18)	0.7500	0.01981 (12)	
O1	1.00329 (10)	0.31848 (9)	0.52371 (16)	0.0310 (3)	
O2	1.18776 (11)	0.27709 (9)	0.47076 (14)	0.0347 (3)	
O3	0.86671 (12)	0.23178 (8)	0.74938 (13)	0.0291 (3)	
H3Y	0.8214	0.2383	0.8237	0.044*	
H3X	0.8264	0.2356	0.6701	0.044*	

### Atomic displacement parameters (Å^2^)

**Table:**

	*U*^11^	*U*^22^	*U*^33^	*U*^12^	*U*^13^	*U*^23^
C1	0.0277 (8)	0.0378 (10)	0.0358 (10)	0.0029 (7)	−0.0078 (8)	−0.0101 (8)
C2	0.0317 (9)	0.0328 (9)	0.0414 (11)	0.0050 (8)	−0.0073 (8)	−0.0121 (8)
C3	0.0260 (8)	0.0294 (9)	0.0327 (9)	0.0065 (7)	−0.0078 (7)	−0.0043 (7)
C4	0.0341 (10)	0.0268 (9)	0.0617 (14)	0.0004 (7)	−0.0200 (10)	−0.0014 (9)
C5	0.0221 (8)	0.0304 (9)	0.0344 (9)	0.0059 (6)	−0.0081 (7)	−0.0022 (7)
C6	0.0358 (10)	0.0244 (8)	0.0621 (14)	0.0050 (7)	−0.0197 (10)	0.0031 (9)
C7	0.0272 (8)	0.0337 (9)	0.0189 (7)	0.0008 (7)	−0.0008 (6)	−0.0015 (7)
C8	0.0321 (10)	0.0489 (11)	0.0233 (8)	0.0010 (9)	0.0014 (7)	0.0001 (8)
N1	0.0212 (7)	0.0307 (8)	0.0266 (7)	0.0027 (6)	−0.0046 (5)	−0.0025 (6)
N2	0.0230 (7)	0.0287 (7)	0.0346 (8)	0.0049 (6)	−0.0086 (6)	−0.0031 (6)
Ni1	0.01593 (17)	0.02772 (18)	0.01579 (17)	0.000	−0.00177 (9)	0.000
O1	0.0255 (6)	0.0512 (9)	0.0164 (6)	0.0054 (5)	−0.0009 (4)	−0.0025 (5)
O2	0.0270 (6)	0.0532 (8)	0.0238 (6)	0.0093 (6)	0.0004 (5)	−0.0013 (6)
O3	0.0263 (6)	0.0357 (7)	0.0253 (7)	−0.0042 (5)	−0.0021 (5)	−0.0035 (5)

### Geometric parameters (Å, º)

**Table:**

C1—C2	1.353 (3)	C6—H6	0.9300
C1—N1	1.381 (2)	C7—O1	1.253 (2)
C1—H1	0.9300	C7—O2	1.255 (2)
C2—N2	1.373 (2)	C7—C8	1.496 (3)
C2—H2	0.9300	C8—C8^ii^	1.326 (4)
C3—N1	1.310 (2)	C8—H8	0.9300
C3—N2	1.348 (2)	N1—Ni1	2.0670 (15)
C3—H3	0.9300	Ni1—O1	2.0443 (15)
C4—C5	1.379 (3)	Ni1—O1^iii^	2.0443 (15)
C4—C6^i^	1.381 (3)	Ni1—N1^iii^	2.0671 (15)
C4—H4	0.9300	Ni1—O3^iii^	2.1247 (13)
C5—C6	1.375 (3)	Ni1—O3	2.1247 (13)
C5—N2	1.425 (2)	O3—H3Y	0.8500
C6—C4^i^	1.381 (3)	O3—H3X	0.8501
			
C2—C1—N1	109.66 (16)	C3—N1—Ni1	123.47 (13)
C2—C1—H1	125.2	C1—N1—Ni1	130.81 (12)
N1—C1—H1	125.2	C3—N2—C2	107.19 (15)
C1—C2—N2	106.02 (17)	C3—N2—C5	125.53 (15)
C1—C2—H2	127.0	C2—N2—C5	127.28 (15)
N2—C2—H2	127.0	O1—Ni1—O1^iii^	175.41 (8)
N1—C3—N2	111.46 (16)	O1—Ni1—N1	89.42 (5)
N1—C3—H3	124.3	O1^iii^—Ni1—N1	93.80 (5)
N2—C3—H3	124.3	O1—Ni1—N1^iii^	93.79 (5)
C5—C4—C6^i^	119.07 (18)	O1^iii^—Ni1—N1^iii^	89.42 (5)
C5—C4—H4	120.5	N1—Ni1—N1^iii^	91.04 (9)
C6^i^—C4—H4	120.5	O1—Ni1—O3^iii^	87.80 (5)
C6—C5—C4	120.85 (16)	O1^iii^—Ni1—O3^iii^	88.96 (5)
C6—C5—N2	119.55 (16)	N1—Ni1—O3^iii^	177.19 (5)
C4—C5—N2	119.57 (16)	N1^iii^—Ni1—O3^iii^	89.51 (6)
C5—C6—C4^i^	120.07 (18)	O1—Ni1—O3	88.96 (5)
C5—C6—H6	120.0	O1^iii^—Ni1—O3	87.79 (5)
C4^i^—C6—H6	120.0	N1—Ni1—O3	89.50 (6)
O1—C7—O2	126.58 (16)	N1^iii^—Ni1—O3	177.19 (5)
O1—C7—C8	116.29 (16)	O3^iii^—Ni1—O3	90.09 (8)
O2—C7—C8	117.06 (16)	C7—O1—Ni1	130.74 (12)
C8^ii^—C8—C7	122.8 (2)	Ni1—O3—H3Y	109.6
C8^ii^—C8—H8	118.6	Ni1—O3—H3X	109.3
C7—C8—H8	118.6	H3Y—O3—H3X	109.5
C3—N1—C1	105.67 (15)		
			
N1—C1—C2—N2	0.8 (2)	C4—C5—N2—C2	36.9 (3)
C6^i^—C4—C5—C6	0.6 (4)	C3—N1—Ni1—O1	45.30 (16)
C6^i^—C4—C5—N2	178.9 (2)	C1—N1—Ni1—O1	−131.70 (17)
C4—C5—C6—C4^i^	−0.6 (4)	C3—N1—Ni1—O1^iii^	−131.42 (15)
N2—C5—C6—C4^i^	−178.9 (2)	C1—N1—Ni1—O1^iii^	51.58 (17)
O1—C7—C8—C8^ii^	−16.9 (2)	C3—N1—Ni1—N1^iii^	139.09 (17)
O2—C7—C8—C8^ii^	160.34 (12)	C1—N1—Ni1—N1^iii^	−37.91 (15)
N2—C3—N1—C1	−0.2 (2)	C3—N1—Ni1—O3^iii^	38.0 (12)
N2—C3—N1—Ni1	−177.88 (12)	C1—N1—Ni1—O3^iii^	−139.0 (10)
C2—C1—N1—C3	−0.4 (2)	C3—N1—Ni1—O3	−43.66 (15)
C2—C1—N1—Ni1	177.04 (15)	C1—N1—Ni1—O3	139.33 (17)
N1—C3—N2—C2	0.7 (2)	O2—C7—O1—Ni1	2.1 (3)
N1—C3—N2—C5	−179.97 (17)	C8—C7—O1—Ni1	179.05 (13)
C1—C2—N2—C3	−0.9 (2)	O1^iii^—Ni1—O1—C7	−73.40 (17)
C1—C2—N2—C5	179.80 (19)	N1—Ni1—O1—C7	152.04 (17)
C6—C5—N2—C3	36.0 (3)	N1^iii^—Ni1—O1—C7	61.04 (17)
C4—C5—N2—C3	−142.3 (2)	O3^iii^—Ni1—O1—C7	−28.32 (17)
C6—C5—N2—C2	−144.8 (2)	O3—Ni1—O1—C7	−118.44 (17)

Symmetry codes: (i) −*x*+1, −*y*+1, −*z*+1; (ii) −*x*+2, *y*, −*z*+1/2; (iii) −*x*+2, *y*, −*z*+3/2.

### Hydrogen-bond geometry (Å, º)

**Table:**

*D*—H···*A*	*D*—H	H···*A*	*D*···*A*	*D*—H···*A*
O3—H3*Y*···O2^iii^	0.85	1.96	2.7033 (18)	146
O3—H3*X*···O2^iv^	0.85	2.03	2.8361 (18)	159
C3—H3···O2^iv^	0.93	2.49	3.360 (2)	155

Symmetry codes: (iii) −*x*+2, *y*, −*z*+3/2; (iv) *x*−1/2, −*y*+1/2, −*z*+1.

## References

[bb1] Batten, S. R. & Robson, R. (1998). *Angew. Chem. Int. Ed.* **37**, 1460–1494. 10.1002/(SICI)1521-3773(19980619)37:11<1460::AID-ANIE1460>3.0.CO;2-Z 29710936

[bb2] Brandenburg, K. (2010). *DIAMOND* Crystal Impact GbR, Bonn, Germany.

[bb3] Bruker (2003). *SAINT* Bruker AXS Inc., Madison, Wisconsin, USA.

[bb4] Bruker (2004). *APEX2* Bruker AXS Inc., Madison, Wisconsin, USA.

[bb5] Bu, X. H., Tong, M. L., Chang, H. C., Kitagawa, S. & Batten, S. R. (2004). *Angew. Chem. Int. Ed.* **43**, 192–195. 10.1002/anie.200352024 14695606

[bb6] Carlucci, L., Ciani, G. & Proserpio, D. M. (2003*a*). *Coord. Chem. Rev.* **246**, 247–289.

[bb7] Carlucci, L., Ciani, G. & Proserpio, D. M. (2003*b*). *CrystEngComm*, **5**, 269–279.

[bb8] Chen, S. S., Bai, Z. S., Fan, J., Lv, G. C., Su, Z., Chen, M. S. & Sun, W. Y. (2010). *CrystEngComm*, **12**, 3091–3104.

[bb9] Harriman, A. & Sauvage, J. P. (1996). *Chem. Soc. Rev.* pp. 41–48.

[bb10] Li, Y. W., Ma, H., Chen, Y. Q., He, K. H., Li, Z. X. & Bu, X. H. (2012). *Cryst. Growth Des.* **12**, 189–196.

[bb11] Moulton, B. & Zaworotko, M. J. (2001). *Chem. Rev.* **101**, 1629–1658. 10.1021/cr9900432 11709994

[bb12] Raymo, F. M. & Sauvage, J. P. (1999). *Chem. Rev.* **99**, 1643–1664. 10.1021/cr970081q 11849006

[bb13] Sheldrick, G. M. (1996). *SADABS* University of Göttingen, Germany.

[bb14] Sheldrick, G. M. (2008). *Acta Cryst.* A**64**, 112–122. 10.1107/S0108767307043930 18156677

[bb15] Sun, D. F., Ma, S. Q., Ke, Y. X., Collins, D. J. & Zhou, H. C. (2006). *J. Am. Chem. Soc.* **128**, 3896–3897. 10.1021/ja058777l 16551082

[bb16] Westrip, S. P. (2010). *J. Appl. Cryst.* **43**, 920–925.

[bb17] Wu, H., Liu, H. Y., Liu, B., Yang, J., Liu, Y. Y., Ma, J. F., Liu, Y. Y. & Bai, H. Y. (2011). *CrystEngComm*, **13**, 3402–3407.

